# Spatiotemporal Analysis of AIDS Incidence and Its Influencing Factors on the Chinese Mainland, 2005–2017

**DOI:** 10.3390/ijerph18031043

**Published:** 2021-01-25

**Authors:** Yige Wang, Chunhong Zhao, Ziping Liu, Decai Gao

**Affiliations:** Key Laboratory of Geographical Processes and Ecological Security of Changbai Mountains, Ministry of Education, School of Geographical Sciences, Northeast Normal University, Changchun 130000, China; wangyg503@nenu.edu.cn (Y.W.); zhaoch341@nenu.edu.cn (C.Z.); liuzp075@nenu.edu.cn (Z.L.)

**Keywords:** spatial analysis, Geographically Weighted Regression, AIDS, spatial epidemiology, Moran’s I index, Markov chain

## Abstract

Acquired Immune Deficiency Syndrome (AIDS) has become one of the most severe public health issues and nowadays around 38 million people are living with the human immunodeficiency virus (HIV). Ensuring healthy lives and promoting well-being is one of 17 United Nations Sustainable Development Goals. Here, we used the Markov chain matrix and geospatial clustering to comprehensively quantify the trends of the AIDS epidemic at the provincial administrate level in the mainland of China from 2005 to 2017. The Geographically Weighted Regression (GWR) model was further adopted to explore four groups of potential influencing factors (i.e., economy, traffic and transportation, medical care, and education) of the AIDS incidence rate in 2017 and their spatially distributed patterns. Results showed that the AIDS prevalence in southeastern China had been dominant and become prevalent in the past decade. The AIDS intensity level had been increasing between 2008 and 2011 but been gradually decreasing afterward. The analysis of the Markov chain matrix indicated that the AIDS epidemic has been generally in control on the Chinese mainland. The economic development was closely related to the rate of AIDS incidence on the Chinese mainland. The GWR result further suggested that medical care and the education effects on AIDS incidence rate can vary with different regions, but significant conclusions cannot be directly demonstrated. Our findings contribute an analytical framework of understanding AIDS epidemic trends and spatial variability of potential underlying factors throughout a complex extent to customize scientific prevention.

## 1. Introduction

Human immunodeficiency virus (HIV)/acquired immune deficiency syndrome (AIDS) has become one of the most severe public health issues since the first cases of AIDS were identified in 1981 [1]. At the end of 2019, approximately 38.0 million (31.6 million–44.5 million) people are living with HIV [2]. Due to gaps in HIV services, about 32.7 million (24.8 million–42.2 million) people have died from AIDS-related illnesses [2]. Ensuring healthy lives and promoting well-being for all ages were addressed by the United Nations as one of 17 Sustainable Development Goals in the 2030 Agenda [3]. Currently, the research on AIDS is mostly focused on epidemiology, and comparative analysis between different social characteristics and geographical populations has been done by epidemiologists [4]. Since the generation, transmission, and distribution of AIDS are closely related to geospatial information [5], tabular records could lose critical information for vaccination tracking and surveillance due to the neglect of the spatial and geographic attributes related to AIDS cases.

As a branch of epidemiology, spatial epidemiology has developed rapidly in recent years. With spatial statistical methods, spatial epidemiology improves the understanding of disease from qualitative level to quantitative level, by analyzing geographically indexed health data related to demography, environment, behavior, socio-economy, genetics, etc. [6,7]. Supported by geographic information science (GIS) and spatial analysis technology, spatial epidemiology describes and analyzes the spatial distribution characteristics and development pattern of epidemiological health events. With a vivid summary and precise pattern of areas at apparently high risk [8], the related disease maps can be used for various descriptive purposes, including aid policy formation and resource allocation [6,9].

Spatial analysis has become increasingly common in HIV/AIDS-related research, specifically for the geographic distribution assessment [10]. For instance, Bautista et al. [11] analyzed the spatial distribution of HIV infection among U.S. civilian applicants for US military service from 1985 to 2003, and found the difference in HIV spatial aggregation between white people and African Americans. Focusing on HIV infection rate in rural areas of South Africa, Tanser et al. provided clear empirical evidence for the localized clustering of HIV infection [12]. The study detected considerable geographical variation in local HIV prevalence within relatively homogenous population, i.e., population do not have much geographical socioeconomic variation. In a recent study of the prevalence of HIV in sub-Saharan Africa, Dwyer-Lindgren et al. [13] revealed considerable within-country variation and local differences in the change direction and rate in HIV prevalence from 2000 to 2017, which brought attention to the subnational variation in HIV prevalence and the related fine-scale estimation and interventions. In China, trend surface analysis and spatial autocorrelation analysis on AIDS have been conducted for a single province or city, including Yunnan [14] and Lanzhou [15]. However, most publications have merely addressed characteristics of the HIV infection at the regional level.

For truly controlling HIV and directly reducing the number of new infections, an understanding of the spatial distribution needs to be combined with an investigation of potential influencing factors. Smith et al. [16] found that education level is an important factor in AIDS risk in rural Uganda. Liu et al. [17] studied the spatial distribution and influencing factors of HIV-infected people in Henan Province, and found that economic level and medical level will have a certain impact on the incidence of AIDS. Most recently, Yang and Li [18] found that the economy, medical treatment, and transportation (i.e., population flow) have an impact on the AIDS incidence in China. Conventional statistics (e.g., ordinary least squares (OLS)) are the primary tools to examine the impacts of underlying factors. However, the prominent limitation of conventional statistics in geoscience is the incapability in capturing the spatial non-stationarity, i.e., spatially varying relationships between dependent and independent variables [19,20]. Moreover, OLS has been shown to be of limited utility when spatial data are coupled with highly correlated independent variables [21,22,23]. An alternative to conventional statistics is Geographically Weighted Regression (GWR).

The discipline of the spatial and temporal analysis of the AIDS incidence and its influencing factors are not uniform. So far, the research mainly focuses on the analysis of AIDS spatial distribution, especially in South Africa [9,12,13,24,25], At present, the related research on AIDS in China mainly focuses on the analysis of epidemiological characteristics [26], prevention and control [27,28], the epidemic situation of the specific population [29,30], etiology and sociology [18,31], however, there are few studies on the spatial-temporal change analysis and influencing factors analysis. The long time series study on AIDS incidence, the future trend, and factors affecting AIDS spread in China have not yet explored.

To fill the gap, this study collected the AIDS incidence data on Chinese mainland from 2005 to 2017 to study the spatial distribution characteristics and influencing factors of the AIDS epidemic, so as to provide a theoretical basis for policymakers to customize scientific prevention and control policies. The study focused on AIDS incidence, i.e., the body’s immune system is badly damaged because of the HIV virus. The paper is organized as follows. After the description of the research data and sources, Section 2 presents the mean research methods, including AIDS intensity level mapping (Section 2.1), Markov chain matrix analysis (Section 2.2), Spatial clustering character (Section 2.3), and GWR analysis (Section 2.4). The related results are followed in Section 3. Section 4 discusses key issues of the AIDS intensity on the Chinese mainland, and the findings based on the analytical method are also summarized. Finally, short conclusions are provided in Section 5.

## 2. Methods

### 2.1. Data Collection

AIDS incidence data was acquired from the Data Center of China Public Health Science (www.phsciencedata.cn). The AIDS prevalence in China for the period 2005–2017 was investigated, considering the data resource consistency and the fact that AIDS was drawn attention by the Chinese government around the year 2005 [32]. The personal information of the individual participant had already been taken away before the release for privacy protection. The study area includes 31 provincial administrative units on the Chinese mainland.

The selection of the potential underlying factors was guided by expert knowledge from wide studies and publications on the epidemic and infectious diseases. Considering the variables type and their relationship with the AIDS infection, the explanatory factors were classified into four categories: economy, traffic and transportation, medical care, and education. First, in most rural areas of China, the economic level is widely regarded as an important factor affecting people’s medical treatment and medical examination affordability. In addition, population flow [33,34] and drug trafficking [35] are important factors in the spread of AIDS and HIV diagnosis and prevention. In this sense, we chose passenger volume, highway length, overall passenger flow, and vehicle ownerships as indicators to measure the level of traffic development. Third, medical and health resources are an important means to deal with epidemic diseases, and differences in access to health care, treatment modalities, and outcomes among different ethnic, and ethnic groups have been confirmed [9]. Finally, education is a notable factor of the spread of epidemiology [16,36,37], especially in rural areas. Meanwhile, residents with lower education have a lower chance to receive diagnostic and medical checks without awareness and required knowledge [38]. The above-mentioned influencing factors are all from *China Statistical Yearbook*, as summarized in Table A1 in Appendix A.

### 2.2. AIDS Intensity Level Mapping

To understand the AIDS intensity level for individual provincial administrative units, the AIDS rate for each year were first calculated:

(1) $$AIDS\_Rate_{ij}=\frac{The\;number\;of\;AIDS\;patients\;in\;the\;administrative\;unit\;i\;for\;the\;year\;j\;}{\mathrm{Population}\;in\;the\;administrative\;unit\;\;i\;for\;the\;year\;j\;}$$

where i refers to the provincial administrative unit ID (ranging from 1 to 31 modified from the national standard geocodes at the provincial level), and *j* refers to the study year with a range from 2005 to 2017.

To make visual comparisons in terms of AIDS intensity among individual provincial administrative units and between different study time points, we further calculated the distribution frequency of AIDS intensity for each individual provincial administrative unit:

(2) $$AIDS\_{\mathrm{Intensity}}_{ij}=\frac{AIDS\_Rate_{ij}}{\sum_{i=1}^{31}AIDS\_Rate_{ij}/31}$$

where $\sum_{i=1}^{31}AIDS\_Rate_{ij}/31$ is overall AIDS incidence intensity for year *j* for the entire Chinese mainland. $AIDS\_{\mathrm{Intensity}}_{ij}$ refers to AIDS intensity for individual provincial administrative unit *i* for year *j*. An $AIDS\_{\mathrm{Intensity}}_{ij}$ value of 1 represents an average intensity of the administrative unit *i* to the overall AIDS intensity for the Chinese mainland. Based on it, the AIDS intensity levels for the administrative unit *i* in year *j* were further categorized (Table 1).

### 2.3. Markov Chain Matrix Analysis

A Markov chain is defined as a process with a limited number of states with the Markovian property and some transition probabilities [36]. A Markov chain can either remain the same or transit to the other state for a time period in a Markov chain process. The length of a Markov chain is the segmentation times between two successive observations. For our AIDS intensity study, there are four states (i.e., intensity levels) for each administrative unit. In other words, one of the four values (1, 2, 3, and 4) can be assigned to the variable of AIDS intensity level in the series at each time t, corresponding to a different state. Given that the transition between two successive states only depends on the AIDS intensity level at the starting time, a first-order time dependence 4-state Markov chain was constructed (Table 2).

There are 4 × 4 transition probabilities (*m_ij_*) for our Markov chain illustrated in the Table 2. The diagonal of the matrix (*m_ij_*, I = *j*) is a smooth transition, i.e., the state of AIDS intensity level is stable for one Markov chain process. The transition probabilities in the upper-right corner (*m_ij_*, I < *j*) refers to an upward transition, and the transition probabilities in the lower-left corner (*m_ij_*, I > *j*) refers to a downward transition. If the state of AIDS intensity level is stable for one Markov chain process, we assume that the AIDS intensity for the corresponding province was in control. An upward transition for the AIDS intensity level indicates that the AIDS epidemic situation was serious and a download transition for the AIDS intensity level indicates that the AIDS epidemic situation was reduced. The calculation formula of each element *m_ij_* in the matrix is:

(3) $$m_{ij}=\frac{n_{ij}}{N_{i}}$$

where $m_{ij}$ represents the probability of a type *i* AIDS intensity level (i.e., code number) transforming to a type *j* AIDS intensity level; $n_{ij}$ is the sum of the number of units converted from type i into type j for all the segmentation times during one Markov chain study period; and $N_{i}$ is the sum of the number of units belonging to type i for the segmentation times during one Markov chain study period.

### 2.4. Spatial Clustering Character of AIDS Incidence

In this study, we assume that the spatial patterns of AIDS were not randomly distributed, since AIDS infections are related to the interactions between cases in different places. In order to explore the spatial clustering and heterogeneity character of AIDS incidence rate for the entire Chinese mainland, the spatial autocorrelation analysis (Global Moran’s I) was conducted. Spatial autocorrelation can be used to describe the spatial difference between the spatial units and their adjacent units. Moran Index was proposed in 1950 [37] and have been intensively applied to determine spatial autocorrelation character. Here, the Global Moran Index measure for clustering (positive spatial autocorrelation) or dispersion (negative spatial autocorrelation) of the AIDS incidence. A Moran’s Index value near 1 indicates clustering, whereas a value near −1 indicates dispersion.

As a measurement of spatial associations at local level, the Local Indicators of Spatial Autocorrelations (LISA) statistics allow us to detect AIDS clusters on the assumption that the local AIDS spatial pattern was not randomly distributed. Local Moran’s Index tests the agglomeration and difference chrematistics of AIDS incidence rates in different spatial locations. The Local Moran’s I is defined as:

(4) $$Moran^{\prime }s\;I=\frac{n}{S_{0}}*i\frac{\sum_{i}^{n}\sum_{j=1}^{n}w_{ij}\left(x_{i}-\bar{x}\right)\left(x_{j}-\bar{x}\right)}{\sum_{i}^{n}{\left(x_{i}-\bar{x}\right)}^{2}}$$

where *n* is the number of the samples; $x_{i}$ is the attribute value of spatial unit i; $\bar{x}$ is the mean value; $w_{ij}$ is spatial weight; $S_{0}$ is the sum of all elements of the spatial weight matrix. Local Moran’s I analyzes the correlation degree of spatial variables between the observed value and the adjacent space unit, and judges the hot spot area of the spatial object [39]. It ranges from −1 to 1, and the absolute value close to 1 means a high similarity between the tested unit and the adjacent unit (i.e., High-High or Low-Low aggregation) and an value close to 0 indicates a low correlation (i.e., High-Low or Low-High aggregation). In this sense, there are four kinds of combinations between the attribute value of spatial unit *i* and its neighborhood: High-High (H-H), High-Low (H-L), Low-High (L-H), and Low-Low (L-L) aggregation [40]. Local Moran’s I scatter plots for AIDS incidence in different study point were obtained using the GeoDa software environment [41].

Here, AIDS incidence clusters were defined as geographic areas in which AIDS prevalence was disproportionately higher compared to neighboring areas. Hot-cold Spot Analysis (Getis-Ord Gi * statistics) [42] was adopted using the Spatial Analysis toolset in the ArcGIS environment.

### 2.5. GWR Analysis

Unlike OLS, GWR is a spatial regression method to model spatial variation in the relationship between dependent and independent variables and estimates the influence degree [43]. By calculating the local parameters of the regression model, the spatial non-stationarity of each parameter in different spatial ranges can be revealed [44]. The model formula is as follows:

(5) $$y_{i}=\beta_{0}\left(u_{i},v_{i}\right)+\sum_{k=1}^{p}\beta_{k}\left(u_{i},v_{i}\right)x_{ik}+\varepsilon_{i}$$

where: $y_{i}$ is the dependent variable for the location i; $\beta_{0}$ is the intercept, $\left(u_{i},v_{i}\right)$ is the coordinate of the location i, $\beta_{0}\left(u_{i},v_{i}\right)$ is the constant term; $\beta_{k}\left(u_{i},v_{i}\right)$ is the coefficient of the *k*th independent variable of sampling point *i*. Instead of remaining the same everywhere, $\beta_{k}\left(u_{i},v_{i}\right)\;$varies in relation to location *i*, $x_{ik}$ is the *k*th independent variable at location i, $\varepsilon_{i}$ is the random error term at location *i*.

Prior to modeling, correlation analyses were conducted among the potential explanatory variables (Table A1 in Appendix A) to assess the multicollinearity. The correlation analyses indicated that there was a strong multicollinearity issue for variables in the same group. Principal component analysis (PCA) was adopted to eliminate the multicollinearity issue. PCA transforms the group of the potential explanatory variables that may have a correlation into a group of linearly uncorrelated variables by orthogonal transformation [45]. Through data transformation and processing, the potential influencing factors of the AIDS incidence rate can be grouped into less integrated factors, which not only maintains the main information of original factors but also avoid the complexity of the correlation among them.

## 3. Results

**Figure 1:**
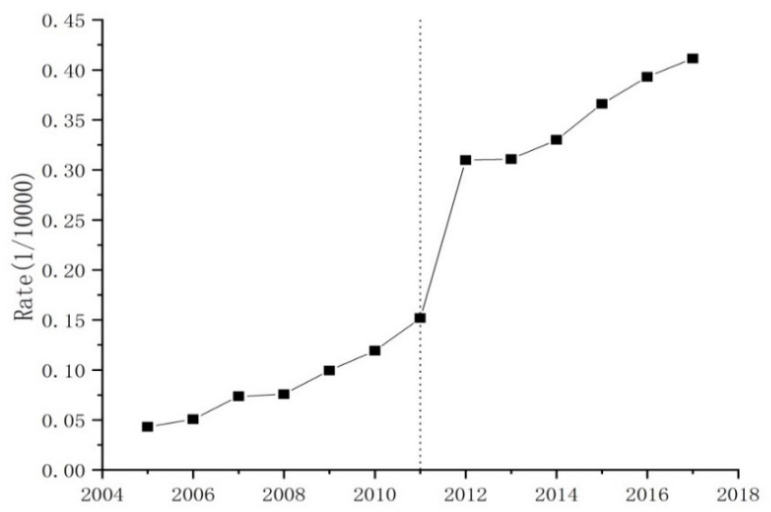
Average AIDS incidence rate trend in the mainland of China during 2005–2017.

The overall change trend AIDS incidence rate at the national level for the mainland of China from 2005 to 2017 can be divided into four stages (Figure 1). The first stage (2005–2008) witnessed a stable growth, with an average AIDS incidence rate of 0.06 per ten thousand persons (0.06/10000). In comparison, the AIDS incidence rate showed a slight increasingly tendency in the second stage (2008–2011), with an average AIDS incidence rate of 0.11 per ten thousand persons (0.11/10000). During the third stage (2011–2014), the AIDS incidence rate exhibited a remarkable fluctuating growth, with an annual growth of 105% from 2011 to 2012 as well as a steady stable growth, with an annual growth of 7.67% from 2012 to 2014. After 2014, the AIDS incidence rate indicated a stable growth again, with an average AIDS incidence rate of 0.38 per ten thousand persons (0.38/10000) and annual growth rate of 11.5%.

### 3.1. Spatiotemporal Variation of AIDS Intensity

**Figure 2:**
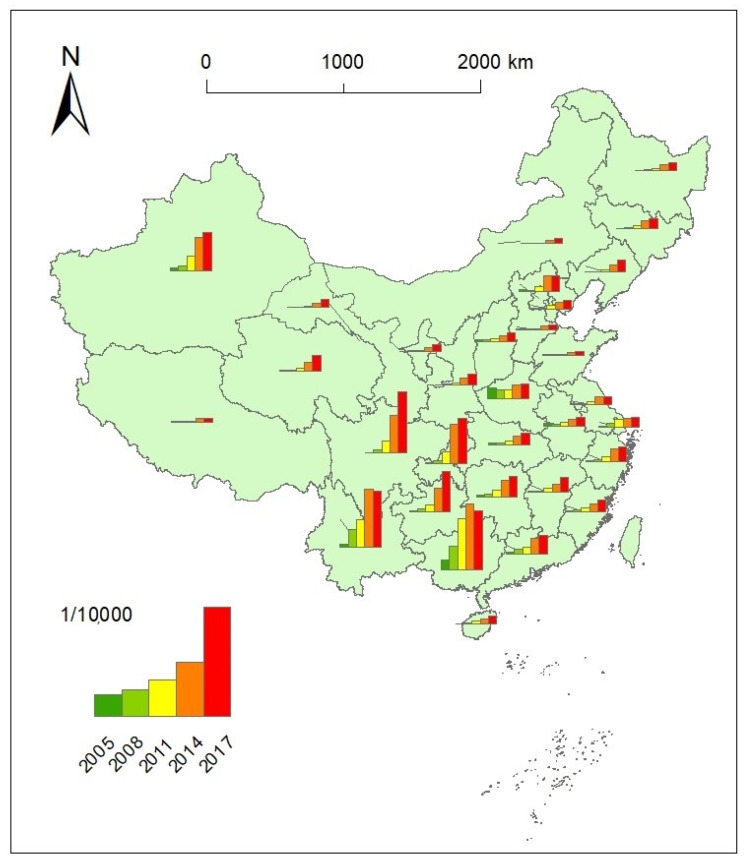
The spatial distribution of AIDS incidence rate on the mainland of China in the year 2005, 2008, 2011, 2014, and 2017.

**Figure 3:**
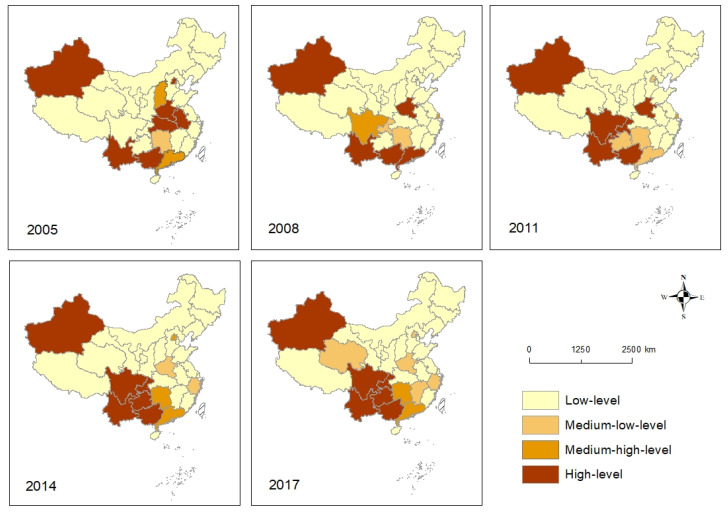
The spatial differences and evolution of AIDS intensity levels in the mainland of China from 2005 to 2017.

The spatial distribution of AIDS incidence rates at the provincial scale was first visualized intuitively in ArcGIS10.2 software (ERSI, Redlands, USA, Figure 2 and Figure 3). The AIDS incidence rate had been increasing for most of the provincial units on the Chinese mainland (Figure 2). Apparently, the differences in AIDS incidence rates were very high during the entire study period, with high incidence rates in southwestern China, including Yunnan, Guangxi, Guizhou, Sichuan and Chongqing, and Xinjiang in northeastern China. Meanwhile, there were considerable differences among these provincial administrative units in the AIDS incidence rate from 2014 to 2017, and the AIDS incidence rate showed a decreasing tendency for Guangxi and Yunnan. In contrast, the AIDS incidence rates were relatively low, with a generally stable increase during the study period in central and eastern China during the study periods.

The AIDS intensity levels for the individual provincial administrative unit for the above time points were further calculated (Table A2 in the Appendix A). The AIDS intensity level charts in 2005, 2008, 2011, 2014, and 2017 showed the difference in the spatial distribution and temporal variation (Figure 3). Different from other periods, the high AIDS intensity levels were relatively scattered in space in 2005. Yunnan, Guangxi, Beijing, Xinjiang, and three adjacent provinces in central China showed high AIDS intensity levels in 2005. From 2008 to 2017, the high incidence areas gradually monopolized in the southwestern region, and the spatial agglomeration was enhanced. The spatial pattern of AIDS intensity levels changed greatly from 2005 to 2008. The AIDS high-value zone expanded to Sichuan and Chongqing and the pattern of AIDS low-level intensity areas remained relatively stable from 2008 to 2011. There is a note that the AIDS intensity level changed from high (code 1) to the middle and low level (code 3) for Henan from 2011 to 2014. The spatial pattern of AIDS high-level intensity areas remained relatively stable from 2014 to 2017.

Spatial Markov matrixes were calculated for the respective four stages to compare the AIDS intensity level evolution character classified above (Table 3). Notably, smooth transition probabilities of low-low ($m_{11}$) and high-high ($m_{44}$) AIDS intensity levels were above 0.86, and the highest was 1 for the four stages, which were far higher than the average level of other transition probability. It is also noted that the transition probabilities between adjacent levels were much greater than that between cross levels, indicating that the temporal evolution of AIDS intensity are gradual rather than jumping. The transition probabilities related to medium-high levels and medium-low levels transiting to other levels were relatively unstable, ranging from 0.17 to 1.

In terms of the AIDS intensity evolution characteristics between different stages, the overall downward transition probabilities between 2005 and 2008 were higher than the upward transition probabilities, indicating that the AIDS intensity level gradually decreased; the upward transfer probabilities between 2008 and 2011 were greater than the downward transfer probabilities, indicating that AIDS risk had been gradually increasing. In the next two stages, the upward transition probabilities had been decreasing from 0.3 to 0.03 and the downward transition probabilities had been decreasing from 0.4 to 0.01, indicating that AIDS risk had been gradually decreasing.

### 3.2. Spatial Agglomeration Characteristic of AIDS

Table A3 in Appendix A provides the Global Moran’s I index statistical result for the AIDS incidence rates. In the year 2005, 2006, and 2007, the returned *p*-value is not statistically significant, and the global spatial autocorrelation characteristics of the AIDS incidence rates was not detected. Started from 2009, *p* values were all <0.05 (passed through the 95% confidence test) and z scores are positive (>1.65, the threshold set by the null hypothesis being rejected), indicating that the AIDS incidence rates had become spatially clustered. Especially, between 2011 and 2017, the global Moran’s I index raised rapidly from −0.114 to 0.312, indicating a growing spatial clustering tendency of the AIDS incidence rate for the enter study area.

**Figure 4:**
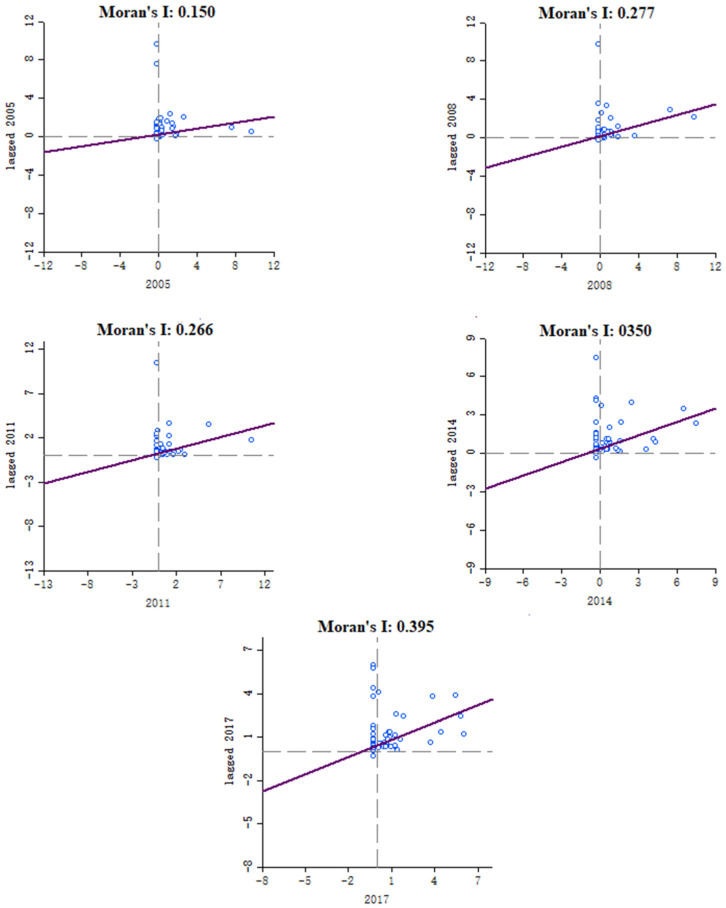
Moran’s I scatter plot of AIDS incidence rates on the Chinese mainland for different time points (2005, 2008, 2011, 2014, and 2017). The trend line passes through the first and third quadrants, so shows high-high aggregation and low-low aggregation.

To further measure the spatial correlation and difference of AIDS incidence rates for each provincial unit and surrounding units, we used the GeoDa software to calculate Moran’s I scatter plots diagrams for five representative time points, as mentioned in Section 3.3. Figure 4 shows that there had been an increasing tendency for Moran’s I of AIDS incidence (0.15, 0.277, 0.266, 0.35, and 0.395) for the five-time points. It is also noted that the scatter points of the five nodes are basically concentrated in the first quadrant, namely H-H aggregation, indicating that there was an obvious spatial aggregation phenomenon in the AIDS high incidence areas.

**Figure 5:**
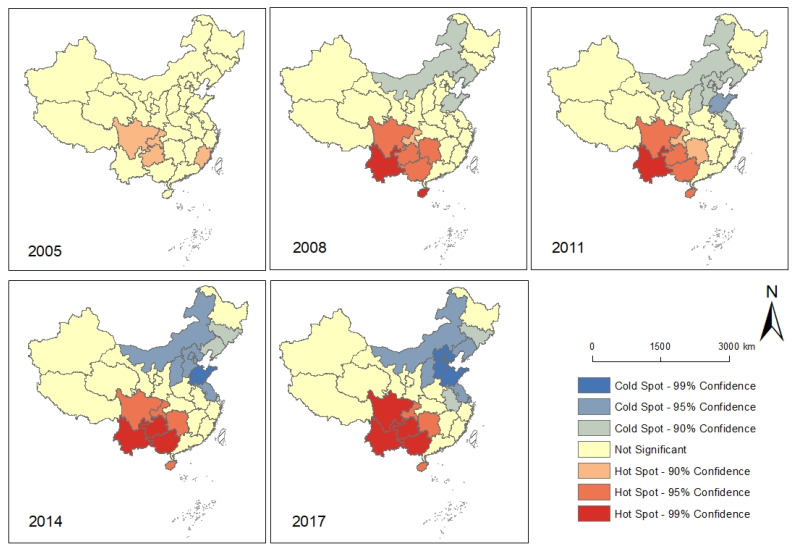
The spatial patterns of the Hot-Cold Spots of AIDS incidence rates in 2005, 2008, 2011, 2014, and 2017.

The Getis-Ord Gi * statistics results at the provincial level were interpreted in the ArcGIS environment. Similar to the interpretation of the Global Moran’s I statistics, the resultant Z score, and *p*-value for each unit determined the high or low values. Based on that, the spatial patterns of the Hot-Cold Spots were illustrated in Figure 5. The spatial agglomeration of hot-cold spots had been gradually enhanced, which was consistent with the global Moran’s index results above. In 2008 and 2011, the Hot Spots of AIDS incidence rates were mainly located at Yunnan, Guangxi, Guizhou, Chongqing, and Sichuan, which were also the Hot Spots in the following two notes with a higher statistical confidence level. Increasingly, starting from 2011, Shandong became the cold spots of AIDS incidence rates with a higher statistical confidence level compared to the sounding provinces, and this cold spot had been expanding continuously with a strong spatial aggregation characteristic. In 2017, the cold spot areas with a 99% confidence level were Shandong, Hebei, Beijing, and Tianjin, and the cold spots also extended to Inner Mongolia, Liaoning, Shaanxi, and Jiangsu with 95% confidence level.

### 3.3. Spatial Pattern of Influencing Factors of AIDS Incidence

**Figure 6:**
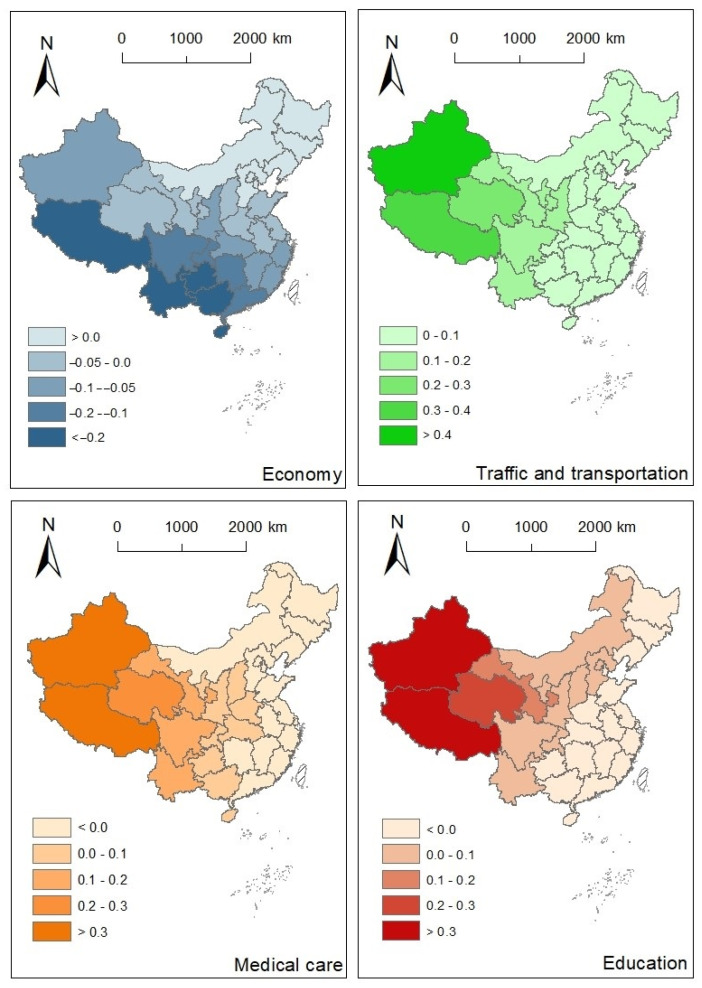
Spatial distribution of regression coefficients: economy, traffic and transportation, medical care and education.

**Figure A1:**
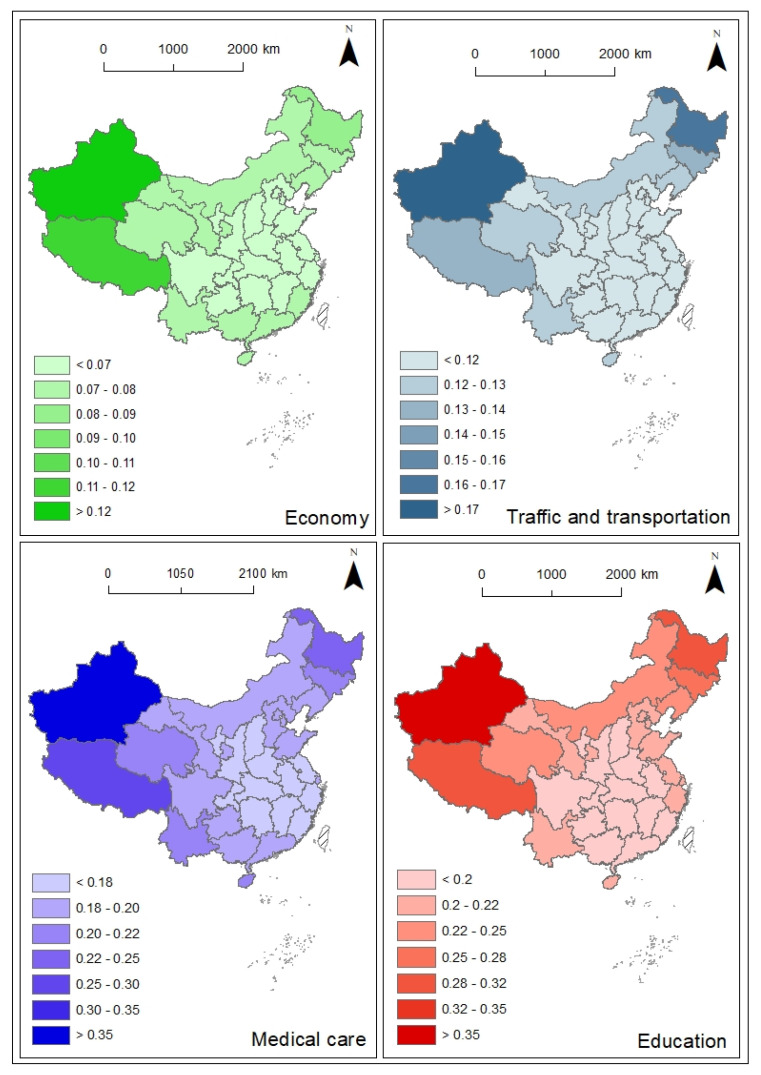
The overall spatial variation of the standard errors for regression coefficients.

For the variables in four groups (i.e., economy, traffic and transportation, medical treatment and education), a comprehensive variable was extracted as the influencing factor of each group of potential explanatory variables. The eigenvalues were 3.79, 1.76, 4.52 and 2.73 respectively, and coefficients were 2.00, 1.46, 2.24 and 1.73 respectively (Table A4 and Table A5 in Appendix A). Taking the AIDS incidence rate in 2017 as the dependent variable, the GWR analysis was conducted on the variables of economy, transportation, medical care, and education level. The distribution of the variables and the homogeneity issue were checked and verified before the regression. The fixed spatial core was selected as Kernel type, and the corrected Akaike Information Criterion (AICc) was selected as the Bandwidth method. The AICc difference of GWR (i.e., 31.2) is much lower than that of the global model (i.e., 33.7), which indicates that GWR provides a better fit to the observed data. Figure 6 and Figure A1 in the appendix showed the overall spatial variation of the GWR coefficients and their standard errors, respectively. For the sake of comparability and interpretation, the influencing variables for each observation were normalized to [0, 1].

For the four variables from different perspectives of socio-economy, the standard errors of coefficients for medical care and education were much higher than those of the other factors. The ratios of the corresponding coefficient value and the standard error were higher than 2.5 for most of the observations (Figure A1 in Appendix B and Figure 6), which indicated that these two variables were not significantly related to the AIDS incidence rate. Conversely, the economy had a generally negative effect on the AIDS incidence rate, based on the coefficient values and their standard error statistics. In comparison, the regression coefficients for traffic and transportation were generally positive and higher than the absolute value of the economy, that is, it had a stronger positive effect on the AIDS incidence rate.

The economy had a negative effect on the AIDS incidence rate for most of the areas in the mainland of China. Relatively absolute higher estimated coefficients were observed in western China (<−0.1) and especially southwestern China (<−0.2). It is noted that the economy level is relatively low in these regions, however, the degree of impacts was relatively large, and attention should also be paid to these areas. It indicated that a lower level of economic development would lead to a decrease in the capacity to prevent and control the AIDS epidemic. An unexpected finding of a slightly positive effect of the economy on the AIDS incidence rate was exhibited in Northeast China. Since the coefficients were near 0, and they were much lower than the coefficients in other regions, the effect of the economy on the AIDS incidence rate is hard to predict. GWR also revealed a strong spatial heterogeneity in the relationships of traffic and transportation and AIDS incidence rate. The more developed, the more the AIDS spread prevalent. The effects of traffic and transportation on the AIDS incidence rate were positive and the coefficient decreased from the west to the east, indicating that the western region is greatly influenced by the traffic and transportation development.

## 4. Discussion

Our results provide a comprehensive quantification of subnational AIDS epidemic trends on the Chinese mainland with geospatial analytical methods. Our study first addressed the spatial variations and time evaluations in AIDS incidence rate and intensity levels. Around the year 2004, the Chinese government and local authorities carried out a series of HIV infection checks and blood tests, to intervene in further HIV transmission through blood [7]. Thus, the AIDS incidence rate had been relatively stable after 2008. The information on AIDS intensity levels “hot spots” of AIDS incidence rate throughout a relatively long period can be used to identify the high risks areas to target primary prevention strategies. For instance, Yunnan is located in the “Golden Triangle” region of Southeast Asia, where drug trafficking is rampant [46]. Our finding revealed that the AIDS prevalence in this region had been dominant and become prevalent in the past decade, which is consistent with the reports that the distribution of HIV/AIDS infection in China is evolving from border regions nearby the “Golden Triangle” to inland areas [47].

Finding on the Markov chain matrix analysis indicated that the high AIDS intensity level area and low AIDS intensity level area tend to maintain the original state in the next period. In other words, these areas had a higher probability of maintaining a high-intensity level in the next coming years and vice versa. Besides, the transfer probability between adjacent two levels is greater than that of cross-level, indicating the space-time transition and evolution had been gradual rather than jumping. Our spatial agglomeration study also demonstrated that a large proportion of AIDS patients had been concentrated in a small number of administrative units with strong spatial correlation. Our analysis highlights this favorable situation to efficiently target resources and interventions for bringing AIDS and HIV infection under control on the Chinese mainland.

Overall, the analysis of GWR revealed that the economy was most closely related to the AIDS incidence rate on the Chinese mainland. First, the economy was shown to be an essential and foremost factor that influences the AIDS incidence pattern. The regression coefficients of economic development were negative except for northeast China, which is consistent with the previous finding related to the spatial distribution of AIDS and main socio-economic driving factors in China [48]. In southwestern China, the economic and education level are relatively low, whereas the sexual transaction and drug abuse situation are severe, thus, more attention should be paid on AIDS and its prevention strategies. In some respects, the situation in the southwestern China confirmed with previous broadly worldwide finding that poverty exacerbates the AIDS/HIV risk [47,49]. The difference in influencing mechanism may also dependent on the cultural backgrounds. Early marriage and childbearing can be another concern for primary AIDS prevention for areas where ethnic minorities gather with a unique culture. Minorities in concentrated communities have a relatively open and tolerant attitude towards sexual behavior and a relatively low awareness of prevention, which increases the possibility of HIV infection. Together with the finding on the spatial clustering tendency for Sichuan, Guizhou, Yunnan, and Guangxi provinces, this area is potentially in need of diagnosis and treatment services.

In terms of the traffic and transportation, the effect was more prevalent in western China than in eastern China, as evidenced by the higher estimated coefficients. The traffic and transportation in this study generally refers to population flow throughout the transportation networks. Even through the transportation by itself do not have a cause on AIDS, since the HIV transmission is different from respiratory epidemics. However, the GWR analysis indicated that the in transnational migrants accompanied by population flow due to trade cooperation and travel, and drug trafficking have a cause on AIDS. Previous study indicated that more than 60% of foreign AIDS cases travel to and from the border between Yunnan and Myanmar, which has also contributed to the spread of AIDS in the region [50]. The frequent cross-border exchanges and interactions is also the cause of the AIDS epidemic in Xinjiang, which is adjacent to the “Golden Crescent”, a drug producing area in Central Asia [51]. There are many drug users in this area and drug users share infected syringes. The population flow due to labor export from rural areas to cities in Sichuan, Chongqing, and Guizhou lead to dangerous sexual behaviors such as multi-sex couples and sex transactions is another susceptible factor for HIV transmission [52]. The evolution of the AIDS incidence levels (Figure 3) and the spatial patterns of AIDS incidence hot spots (Figure 5) might indicate the movement of migrants and an expanded transportation network across the country.

Medical care and education level have been widely identified as important determinants of AIDS prevention at different scales [9,16,36,38]. However, our spatial regression analysis did not indicate a significant correlation at the provincial level. At the local scale, Liu et al. [17] showed that the increase in the number of medical institutions per capita can reduce the HIV infection rate in Henan Province. The improvement of medical care and monitoring systems can enhance the surveillance capability for spatial epidemiology [6]. A recent study of the HIV epidemic among men who have sex with men in China also showed that the number of medical institutions positively correlated with the number of reported HIV/AIDS cases depended on spatial regression at the county level [53].

Previous studies also found that high-educated individuals should be targeted for AIDS prevention [16] and that the individuals with a college degree or above showed a high AIDS incidence rate [53]. Our study showed that the education effects on AIDS incidence rate varied with different regions and significant conclusions cannot be directly demonstrated. In this study, four groups of variables (i.e., economy, traffic and transportation, medical care, and education) were explored as macro comprehensive socio-economic indicators, however, the basic characteristics and information closely related to AIDS incidence may be ignored or missed. The examination of causal relationships of the related factors is needed, since the original selected variables can affect each other [15]. Meanwhile, the overall pattern at the provincial administrative level was estimated, whereas the variations at the local level tend to be masked and the scale issue is an indispensable limitation of this study. The effects of religious beliefs, sex education at high schools can be important factors to intervene HIV transmission. The fine-scale spatiotemporal investigation of AIDS prevalence based on GIS and spatial analysis can be a fundamental method for precise AIDS interventions. Further, we just examined the historical AIDS trends at the provincial administrate level, due to the privacy protection of the AIDS incidence data at local scale. Further investigation of the traffic and transportation for targeted areas may help to figure out the spread of the AIDS in the future.

## 5. Conclusions

In this study, we used a spatially statistical method to explore the spatial distribution of the AIDS epidemic and its influencing factors on the mainland of China from 2005 to 2017. This study first provided a comprehensive quantification of AIDS epidemic trends at the provincial administrate level. According to the analyses of the AIDS intensity level mapping and the spatial clustering, we found that the prevalence of AIDS in southeastern China has remained high in the past decade. The period between 2008 and 2011 witnessed the apparent increasingly severe tendency of the AIDS incidence level, which gradually decreased afterward. Markov chain matrix analysis indicated that AIDS intensity level transfer probability between adjacent two levels was greater than that of cross-level, suggesting the AIDS epidemic has been generally in control on the Chinese mainland. In terms of influencing factors of the AIDS incidence, the economic development closely related to the AIDS incidence rate on the Chinese mainland. The GWR result further showed that the effects of medical care and education on the AIDS incidence rate differed from different regions. Our study contributes to an analytical framework of understanding AIDS epidemic trends and spatial variability of the underlying factors throughout a complex area to customize scientific prevention. In future research, the fine-scale spatiotemporal investigation of AIDS prevalence based on GIS and information related to the spatial variability of relationships can be a fundamental method to precise AIDS interventions.

## Figures and Tables

**Table 1:** The classification scheme for AIDS intensity level for the individual provincial administrative unit.

AIDS Intensity Ranges	AIDS Intensity Level	Intensity Level Coding
$AIDS\_{\mathrm{Intensity}}_{ij}<=0.75$	Low-level	1
$0.75<AIDS\_{\mathrm{Intensity}}_{ij}<=1$	Medium-low-level	2
$1<AIDS\_{\mathrm{Intensity}}_{ij}<=1.25$	Medium-high-level	3
$AIDS\_{\mathrm{Intensity}}_{ij}>1.25$	High-level	4

Note: ${\mathrm{DF}}_{ij}$ is the distribution frequency of AIDS intensity for individual provincial administrative unit *i* for year *j*.

**Table 2:** An example of a Markov transition-probability matrix for the types of AIDS intensity level in a Markov chain process.

Intensity Level (Code) at the Starting Time Point	Intensity Level (Code) at the Ending Time Point			
	Low-Level (1)	Medium-Low-Level (2)	Medium-High-Level (3)	High-Level (4)
Low-level (1)	$m_{11}$	$m_{12}$	$m_{13}$	$m_{14}$
Medium-low-level (2)	$m_{21}$	$m_{22}$	$m_{23}$	$m_{24}$
Medium-high-level (3)	$m_{31}$	$m_{32}$	$m_{33}$	$m_{34}$
High-level (4)	$m_{41}$	$m_{42}$	$m_{43}$	$m_{44}$

Note: *m_ij_* (1 ≤ I ≤ 4; 1 ≤ j ≤ 4) represents the probability of a type -*i* AIDS Intensity Level (code) transforming to a type-*j* AIDS Intensity Level (code) during one study period.

**Table 3:** Spatial Markov matrix (probability) of AIDS intensity level in the four stages: 2005–2008, 2008–2011, 2011–2014, and 2014–2018.

Stage	t/t + 1	Low	Medium-Low	Medium-High	High
2005–2008	Low	0.97	0.02	0.01	0
	Medium-low	0.17	0.33	0.50	0
	Medium-high	0.33	0.33	0.17	0.17
	High	0.04	0.05	0.05	0.86
2008–2011	Low	0.96	0.04	0	0
	Medium-low	0.09	0.55	0.27	0.09
	Medium-high	0	0.50	0.25	0.25
	High	0	0	0.09	0.91
2011–2014	Low	0.99	0.01	0	0
	Medium-low	0.07	0.64	0.22	0.07
	Medium-high	0	0.29	0.71	0
	High	0	0	0.04	0.96
2014–2017	Low	0.97	0.03	0	0
	Medium-low	0	1.00	0	0
	Medium-high	0	0.10	0.90	0
	High	0	0	0	1.00

## Data Availability

We did not upload statistical data but they are available to any author who requests them.

## Appendix A

**Table A1:** Description of a synthesis of potential influencing factors of incidence rates for the mainland of China in 2017.

Variables	Description	Units
**Economy**		
Consumption_level	The ratio of Gross Domestic Product (GDP) consumption to the average annual population.	Yuan
Per_capita_GDP	The ratio of gross domestic products to the total population.	Yuan
Per_capita_income_rural	The gross income of rural households, deducted by the expenses on production, operation, tax payment, and the laboring payment on collective tasks.	Yuan
Per_capita_income_urban	The average wages of urban non-private institutes and urban private institutes.	Yuan
**Traffic and transportation**		
Passenger_volume	The number of passengers actually transported by various types of vehicles of during the year.	10,000 people
Highway_length	The length of highways.	km
Overall_passenger_flow	The product of the number of passengers transported and the transportation distance.	10,000 people
Num_vehicles	The total number of vehicles registered and licensed for civil use.	10,000 vehicles
**Medical care**		
Num_medical_staff	The overall number of staff and related medical workers working in hospitals as well as private medical and health institutions.	1 people
Num_nursing_beds	The fixed actual beds (non-establishment beds), including regular beds, simple beds, monitoring beds, beds under disinfection and repair, etc. The obstetric neonatal beds, delivery room waiting beds, inventory beds, observation beds, temporary additional beds and accompanying beds are excluded.	10,000 beds
Num_medical_insurances	The total number of employees participating in basic medical insurance, i.e., basic medical insurance for urban and rural employees according to relevant government regulations.	10,000
Treatment	The total number of people treated by medical and health institutions.	1,000,000
Num_institutions	Including hospitals, primary medical and health institutions, professional public health institutions and other medical and health institutions.	
**Education**		
Education_funds	The overall public budget for education funds, including taxes and fees collected by governments for all levels for education, funds allocated by enterprises for education in enterprise systems, etc.	10,000 Yuan
Num_high_schools	The national demonstrative ordinary high schools, excluding vocational middle school.	
Num_colleges	The number of full-time universities, independent colleges, etc., recruiting senior high school graduates who passed the National College Entrance Examination.	

**Table A2:** AIDS intensity levels at the individual provincial administrative unit scale for the Chinese mainland for the year 2005, 2008, 2014, and 2017.

Provinces	2005	2008	2011	2014	2017
Beijing	High	Low	Medium-low	Medium-high	Medium-low
Tianjin	Low	Low	Low	Low	Low
Hebei	Low	Low	Low	Low	Low
Shaanxi	Medium-high	Low	Low	Low	Low
Inner Mongolia	Low	Low	Low	Low	Low
Liaoning	Low	Low	Low	Low	Low
Jilin	Low	Low	Low	Low	Low
Heilongjiang	Low	Low	Low	Low	Low
Shanghai	Low	Medium-high	Medium-high	Low	Low
Jiangsu	Low	Low	Low	Low	Low
Zhejiang	Low	Low	Low	Medium-low	Medium-low
Anhui	High	Low	Low	Low	Low
Fujian	Low	Low	Low	Low	Low
Jiangxi	Low	Low	Low	Low	Medium-low
Shandong	Low	Low	Low	Low	Low
Henan	High	High	High	Medium-low	Medium-low
Hubei	High	Low	Low	Low	Low
Hunan	Medium-low	Medium-low	Medium-low	Medium-high	Medium-high
Guangdong	Medium-high	High	Medium-low	Medium-high	Medium-high
Guangxi	High	High	High	High	High
Hainan	Low	Low	Low	Low	Low
Chongqing	Low	Medium-low	High	High	High
Sichuan	Low	Medium-high	High	High	High
Guizhou	Low	Low	Medium-low	High	High
Yunnan	High	High	High	High	High
Xizang	Low	Low	Low	Low	Low
Shanxi	Low	Low	Low	Low	Low
Gansu	Low	Low	Low	Low	Low
Qinghai	Low	Low	Low	Low	Medium-low
Ningxia	Low	Low	Low	Low	Low
Xinjiang	High	High	High	High	High

**Table A3:** The interpretation of Global Moran’s I index statistical result for the AIDS incidence rates in the entire Chinese mainland.

Year	Moran’s I	Expected Index	Variance	Z score	*p* Value
2005	−0.072	−0.033	0.004	−0.602	0.547
2006	−0.017	−0.033	0.005	0.220	0.826
2007	0.034	−0.034	0.004	1.046	0.296
2008	0.083	−0.033	0.004	1.781	0.076
2009	0.092	−0.033	0.004	2.021	0.043
2010	0.095	−0.033	0.004	2.143	0.032
2011	0.114	−0.033	0.004	2.419	0.016
2012	0.160	−0.033	0.005	2.806	0.005
2013	0.202	−0.033	0.005	3.349	0.001
2014	0.223	−0.033	0.005	3.570	0.0004
2015	0.261	−0.033	0.005	4.025	0.00006
2016	0.298	−0.033	0.006	4.461	0.000008
2017	0.312	−0.033	0.006	4.669	0.000003

Note: The Null hypothesis is that the AIDS incidence rates were randomly distributed in the entire Chinese mainland for the respective study time points.

**Table A4:** Results of principal component analysis.

Principal Component	Variables	PC-Loading	Score Coefficient
Economy	Consumption_level	0.98	0.29
	Per_capita_income_urban	0.97	0.26
	Per_capita_GDP	0.97	0.26
	Per_capita_income_rural	0.96	0.25
Traffic and transportation	Passenger_volume	0.93	0.53
	Highway_length	0.78	0.45
	Overall_passenger_flow (10,000)	0.48	0.27
	Num_vehicles	−0.25	−0.14
Medical care	Num_medical_staff	0.99	0.22
	Num_nursing_beds (10,000)	0.98	0.22
	Num_medical_insurances (10,000)	0.97	0.21
	Treatment	0.93	0.21
	Num_institutions	0.89	0.20
Education	Education_funds	0.96	0.35
	Num_high_schools	0.95	0.35
	Num_colleges	0.95	0.35

Note: PC-loadings refers to principal components (PC) loadings. Score coefficient refers to the degree that the case deviates from the mean of all samples.

**Table A5:** Eigenvalues, contribution rates and coefficients of the four principal components.

Principal Component	Eigenvalue	Variance Contribution Rate (%)	PC_Coefficient
PC_Eco	3.79	94.77	2.00
PC_Traff	1.76	80.48	1.46
PC_Med	4.52	90.29	2.24
PC_Edu	2.73	90.90	1.73

PC_Eco refers to the selected principal component of the economy, and so on. Eigenvalue is the contribution of the corresponding eigenvector to the entire matrix, and it is generally greater than 1. The cumulative variance contribution rate is the sum of the variance contribution rates of all principal components, which is generally higher than 80%. Since only one principal component was selected in this study, the variance contribution rate was required to be higher than 80%. The PC_Coefficient refers to the eigenvector of the respective principal component, and the expression is:

(A1) $$\;\;\;\;\;Coefficient_{i}=\sum^{\;}PC\_Loading_{j}/Eigenvalue_{i}$$

where *i* represents different aspects of economy, traffic and transportation, medical care and education, and *j* represents the individual variables in each aspect (Table A4).
